# Evidence for Distinguishable Treatment Costs among Paranoid Schizophrenia and Schizoaffective Disorder

**DOI:** 10.1371/journal.pone.0157635

**Published:** 2016-07-08

**Authors:** Dusan Hirjak, Achim Hochlehnert, Philipp Arthur Thomann, Katharina Maria Kubera, Knut Schnell

**Affiliations:** 1 Center for Psychosocial Medicine, Department of General Psychiatry, University of Heidelberg, Heidelberg, Germany; 2 Center for Psychosocial Medicine, Department of General Internal Medicine and Psychosomatics, University of Heidelberg, Heidelberg, Germany; University of Stellenbosch, SOUTH AFRICA

## Abstract

**Background:**

Schizophrenia spectrum disorders result in enormous individual suffering and financial burden on patients and on society. In Germany, there are about 1,000,000 individuals suffering from schizophrenia (SZ) or schizoaffective disorder (SAD), a combination of psychotic and affective symptoms. Given the heterogeneous nature of these syndromes, one may assume that there is a difference in treatment costs among patients with paranoid SZ and SAD. However, the current the national system of cost accounting in psychiatry and psychosomatics in Germany assesses all schizophrenia spectrum disorders within one category.

**Methods:**

The study comprised a retrospective audit of data from 118 patients diagnosed with paranoid SZ (F20.0) and 71 patients with SAD (F25). We used the mean total costs as well as partial cost, i.e., mean costs for medication products, mean personal costs and mean infrastructure costs from each patient for the statistical analysis. We tested for differences in the four variables between SZ and SAD patients using ANCOVA and confirmed the results with bootstrapping.

**Results:**

SAD patients had a longer duration of stay than patients with SZ (*p* = .02). Mean total costs were significantly higher for SAD patients (*p* = .023). Further, we found a significant difference in mean personnel costs (*p* = .02) between patients with SZ and SAD. However, we found no significant differences in mean pharmaceutical costs (*p* = .12) but a marginal difference of mean infrastructure costs (*p* = .05) between SZ and SAD. We found neither a common decrease of costs over time nor a differential decrease in SZ and SAD.

**Conclusion:**

We found evidence for a difference of case related costs of inpatient treatments for paranoid SZ and SAD. The differences in mean total costs seem to be primarily related to the mean personnel costs in patients with paranoid SZ and SAD rather than mean pharmaceutical costs, possibly due to higher personnel effort and infrastructure.

## Introduction

Schizophrenia (SZ) is a neurodevelopmental disorder characterized by various combinations of delusions, hallucinations, disorganized thoughts and behavior, cognitive impairment and movement disorders [1,2]. The pathophysiology of SZ likely reflects genetic, developmental and environmental factors [3]. The main pathophysiological concept assumes an overstimulation of dopaminergic transmission in striatal, frontal and cingulate brain regions [3,4]. For this reason, the primary pharmacological treatment of patients suffering from SZ involves dopamine antagonists. In addition to antipsychotic medication, psychotherapy and sociotherapy can be instituted to facilitate coping strategies of the patients and for psychosis prophylaxis. According to recent epidemiological studies, 800,000 individuals in Germany are currently afflicted with SZ [5–7]. As the reported lifetime prevalence of SZ is about 1% the disorder constantly causes enormous individual suffering and financial burden on patients and on society [8]. Despite of the clinical delineation of SZ and affective disorders [9], recent neuroscientific research provided evidence for common and disease-specific clinical and biological characteristics of these disorders [10,11].

In 1952, schizoaffective disorder (SAD) as an independent nosological entity was introduced with the first publication of Diagnostic and Statistical Manual of Mental disorders (DSM) [12]. However, the conceptualization and the nosological status of SAD still remain controversial [13]. In the current ICD-10 and DSM-V version, SAD is categorized as a schizophrenia spectrum disorder and requires two clinical syndromes: (1) manifest mood disorder concurrent with (2) at least two of five psychotic symptoms (delusions, hallucinations, disorganized speech, disorganized behavior, negative symptoms) for at least four weeks. The lifetime prevalence for SAD is approximately 0.32% [13,14]. However, there is a paucity of epidemiological studies on the prevalence of SAD. In particular, no epidemiological studies investigated the prevalence of SAD in Germany. A possible explanation might be the low diagnostic reliability and the lack of conceptual consensus of SAD [13].

Because of missing diagnostic biomarkers, schizophrenia spectrum disorders still pose a diagnostic challenge. In daily clinical practice, especially when deciding admission to inpatient treatment it is not uncommon to reserve the diagnosis of SZ for severe cases presenting with flamboyant psychotic symptoms such as bizarre delusions or first-rank symptoms, reflecting uncertainty in distinguishing SZ from SAD. However, patients with SAD might exhibit more severe depressive and negative symptoms, lower age of onset [13], less cognitive disabilities [15], and better social functioning [13] when compared to SZ individuals. Correspondingly, there is some evidence that patients with SAD have better clinical outcome than those with SZ [16]. It is fair to assume that these reflect the episodic character of SAD compared to SZ. Still, the diagnostic stability of SAD is poor and the diagnosis at the first admission is often not definitive [17].

Even in modern psychiatry, the diagnosis of a mental disorder is still based solely on clinical observations and the patient’s narrative. Because of the very heterogeneous presentation of the two disorders, their diagnosis is still a challenge. Correspondingly, their treatment is demanding and a variety of medications can be used to treat both disorders. On the other hand, it is still important to keep the longitudinal overlap of psychotic and affective symptoms in mind and distinguish between these two disorders in order to establish the appropriate combination and dosage of antipsychotic and mood stabilizing medication in SAD. It can be hypothesized that this issue and the remaining diagnostic uncertainty in SAD and the demanded dual treatment strategy is most likely associated with additional clinical effort and resources compared to SZ.

While it is of general interest for psychiatry to test this hypothesis of different resource allocation to SZ and SAD treatments, it is of special importance when economic prerequisites are changing. In Germany, the current lump sum payment scheme for psychiatric services is based on daily treatment costs depending on general categories of human resources allocation in a hospital ward, but independent of patient’s diagnosis. Following the reimbursement in the somatic medicine based on diagnostic related groups (DRGs), in 2009, a new national system of cost accounting in psychiatry and psychosomatics has been introduced. The system is the new basis of daily cost data collection but is still not implemented as the basis of reimbursement. The implementation of this new system as a controlling instrument in the clinical daily routine is currently a very controversial issue.

The main topic is the validity of this diagnosis-related classification within a group of schizophrenia spectrum disorders (ICD 10 F2x.x) which does not map single diagnostic entities but rather groups of diagnoses. However, the variety of clinical subtypes within this major diagnostic domain including schizophrenia, schizotypal and delusional disorders challenges approaches to use such major categories to determine the demand of financial resources in of cost accounting. The distinction of SZ and SAD represents a prototype of dilemma in this discussion, as both disorders are summarized in the same category. However, no studies have investigated the treatment costs between SZ and SAD patients in Germany yet. Along these lines, the first aim of the presented analysis was to explore and compare the duration of treatment, daily treatment costs and overall costs associated with SZ and SAD at the Department of General Psychiatry in Heidelberg. A second issue is the assumption of a decrement in treatment costs presumed by the cost accounting system.

We believe that a comprehensive comparison of the healthcare costs among SZ and SAD patients might provide more insight into the distribution of costs between diagnostic groups and improve the distribution of available treatment resources.

## Methods

### Study participants

We applied a retrospective, non-interventional, cohort study design. The electronic medical record database of the University hospital Heidelberg (ISH-med) was searched for patients who underwent treatment at the Department of General Psychiatry in Heidelberg between 1^st^ January 2013 and 31^st^ December 2013. The Department of General Psychiatry has 127 inpatient beds as well as an outpatient department in a predominantly urban area in Heidelberg and a fully integrated health service providing treatment for patients with all kinds of mental disorders. The retrospective data were examined for daily in-patient costs, duration of treatment and overall residential treatment costs. 189 patients were identified with admission and discharge between 1^st^ January 2013 and 31^st^ December 2013. The inclusion criteria were: (1) confirmed diagnoses of paranoid schizophrenia or SAD and (2) age between 16 and 80 years. The exclusion criteria included incomplete medical histories, diagnosis of comorbid dementia, and serious acute or chronic neurological or internal disorders which would require additional medical treatment. This resulted in 118 patients diagnosed with paranoid schizophrenia (F20.0) and 70 patients with SAD (F25.*) being included in the analysis. One of the main goals of this study was to ensure that the case costs were calculated in a comparable way to previous health-care studies in psychiatry. Therefore, the procedure chosen is according to the categories of the “Institute for the Hospital Remuneration System” (“Institut für das Entgeltsystem im Krankenhaus”—InEK) as described in the Costs Accounting Manual, Version 1.0 [18]. Thus, our comparison of the paranoid SZ and SAD patients complies with a standard method of costing implemented in Germany. According to the *„Costs accounting manual“*, the daily inpatients costs consist of eight cost category groups: (1) personnel costs physician service; (2) personnel costs nursing service; (3) personal costs, medical technical service/function service; (4a) material cost pharmaceutical products; (4b) material costs pharmaceutical products, direct costs; (5) implant and grafts; (6a) material costs; (6b) other medical material costs; (7) personnel and material costs medical infrastructure and (8) personnel and non-material costs medical infrastructure. To provide an overview, we tested for differences between the mean total costs (1–8) as well as differences of partial costs i.e. mean costs for medication products (4a), mean personal costs (1–3) and mean infrastructure costs (7–8) for the statistical analysis. Note that these partial costs do not add up to the amount of total costs, since we did not compare items 4b-6b, which have only very small contributions to the overall costs.

From a clinical perspective, the majority of schizophrenia patients suffer from the paranoid type. In order to exclude patients receiving high-intensity or specialty (e.g., ECT) treatments while on the units, we did not include patients with catatonic, residual, and hebephrenic schizophrenia according to ICD-10. We were able to trace the contribution of medication and personnel costs, but did not include cost based information whether patients received ECT as a special treatment. However, the general frequency of ECT for SZ and SAD in our department was minimal with two patients treated with ECT in 2013. Thus, it is very unlikely that such allocation of resources is a notable contribution to the observed differences.

The cost data are extracted from a validated dataset for cost calculation at the University Hospital in Heidelberg generated for annual economic purposes. The information on daily inpatients costs were obtained by a member of the medical accounting and controlling department who was not directly involved in the study and therefore blind to the hypotheses. Extraction of datasets was performed with modifications of standard reporting scripts of the accounting department. This study used anonymous administrative data without identifiable private information and no human intervention was involved. The study was in accordance with internal ethic guidelines for the use of patients’ data as implemented by the Medical Faculty, University of Heidelberg, Germany.

### Statistical analyses

Data were analyzed using the Statistical Package of the Social Sciences (SPSS version 22.0, SPSS Inc., Chicago, IL). Sociodemographic variables were described and compared between the two groups with unpaired *t-test* or chi-square test for categorical variables using conventional significance levels (*p* < 0.05).

Mean total costs, mean costs for pharmaceutical products, personnel and infrastructure costs as well as the length of stay were analyzed as the sum of daily costs over the duration of treatment (equaling average daily cost x average days). The distribution of individual mean values within these categories fitted to a normal distribution according to the Kolmogorov-Smirnov-Test as implemented in SPSS 22.0. Therefore, the differences in the four variables between the two study groups were assessed using ANCOVA as implemented in SPSS 22.0. Age and gender were included as nuisance variables. In line with the recommendations of Barber et al. [19] and to derive robust estimates of confidence intervals for estimated group differences of mean of total costs, medication costs, personals costs and infrastructure costs, we also performed paired samples t-test with bootstrapping as implemented in SPSS 22.0 (10,000 samples).

In addition to analyzing the group differences of overall costs for the entire inpatient period we tested, if the expected decrement of treatment costs over time was actually present in our data. For all partial categories of costs, i.e. medication, personnel and infrastructure we tested group x time effects and the main effect of time by a repeated measures ANOVA between means of biweekly intervals for weeks 1 to 8 (i.e. averages of week 1–2, 3–4, 5–6, 7–8) where the number of inpatients in both groups was still sufficient to perform such a test. The national costs accounting system actually predicted a decrement reaching a constant level within this interval.

## Results

**Fig 1:**
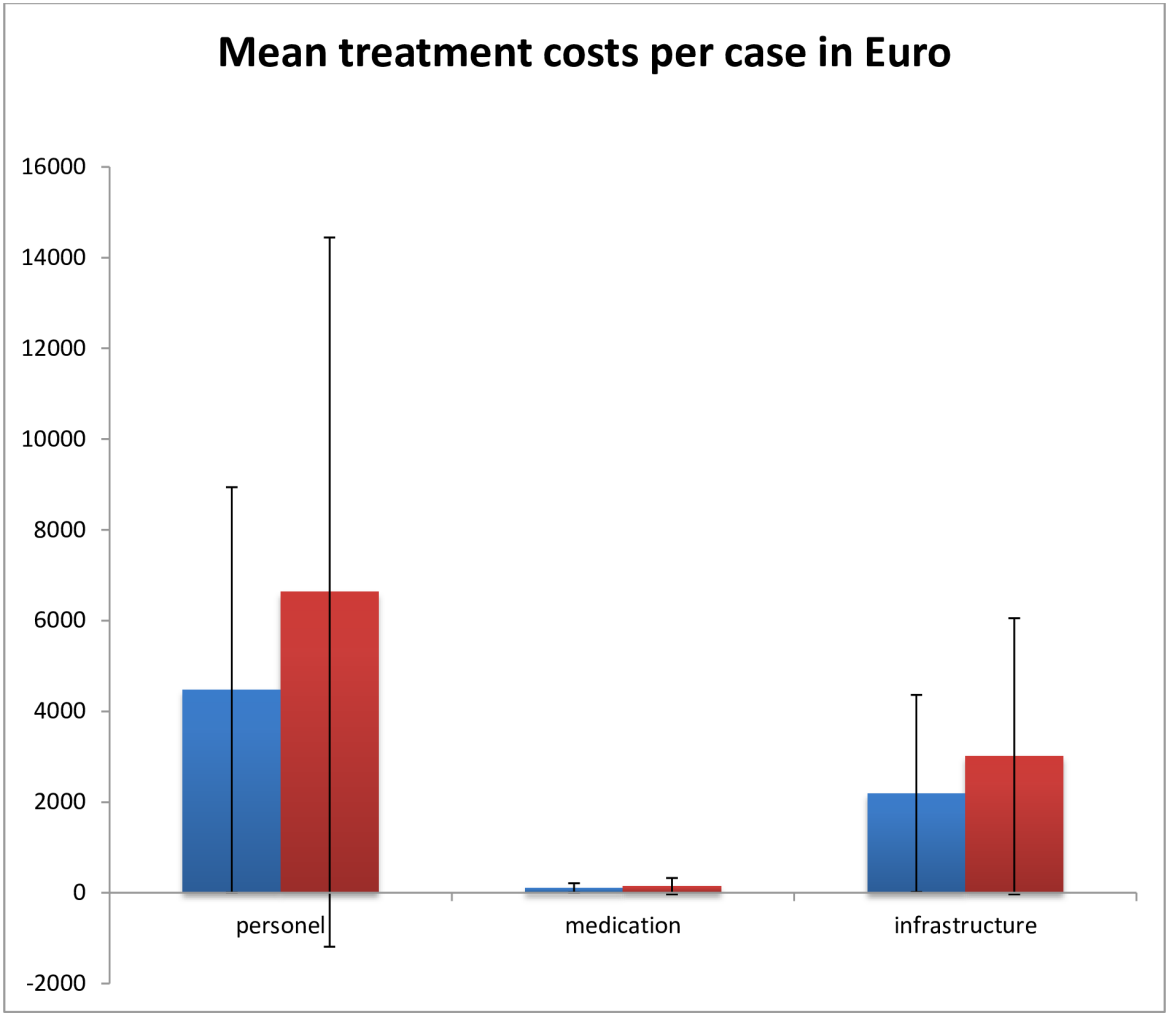
Mean values of personal, medication and infrastructure costs per case in Euro for patients with paranoid SZ (blue) and SAD (red). The ends of the whiskers indicate the standard deviation. Note that these partial costs do not exactly add up to the amount of total costs, since we did not compare costs for materials other than medication.

Demographic characteristics and costs of both clinical groups are summarized in Table 1. Comparison of the two groups revealed no significant difference in gender [chi-square test: χ^2^ = 0.38; df = 1; *p* = 0.846], but a statistically significant difference in age [F (2, 188) = 4.53; *p* < 0.001]. According to ANCOVA, SAD patients had a longer duration of stay than patients with SZ (*p* = .027) (Table 1). Mean total costs were significantly higher for individuals suffering from SAD than paranoid SZ patients [F (1, 185) = 5.28; *p* = .023)] (Fig 1 and Table 1). Further, we found we found statistically significant difference in mean personnel costs [F (1, 185) = 5.21; *p* = .02] between patients with paranoid SZ and SAD. However, we found no statistically significant difference in mean pharmaceutical costs [F (1, 185) = 2.39; *p* = .12)] and a marginal difference of mean infrastructure costs [F (1, 185) = 3.85; *p* = .051] between paranoid SZ and SAD. The costs for medication products, corresponding to item 4a of the German hospital cost accounting algorithm (see above), represented an average of 1.56% of the total costs in patients with paranoid schizophrenia and 1.52% in patients with SAD.

**Table 1:** Demographic characteristics and costs (*n* = 189).

*Characteristics*	*Schizophrenia*	*SAD*	*t-test (bootstrapping)*
**Age (years)**	39.73 ± 12.83	46.94 ± 16.3	0.001
**Male/Female sex**	51/67	41/30	-
**Length of stay (days)**	33.07 ± 30.48	47.69 ± 50.25	**0.027**
**Mean total costs (Euro)**	6769.96±6221.46	9788.57±10652.28	**0.035**
**Mean pharmaceutical costs (Euro)**	105.76±103.67	149.32±180.11	0.067
**Mean personal costs (Euro)**	4472.54±4470.72	6628.92±7816.52	**0.04**
**Mean infrastructure costs (Euro)**	2191.64±2174.75	3010.32±3044.02	**0.053**

Mean and SD are given except when noted.

The paired samples t-test with bootstrapping with 10,000 samples revealed statistically significant differences between the two groups in the mean total costs (p = 0.035), and mean personnel costs (p = 0.04). We found a marginally significant difference in the mean infrastructure costs (p = 0.053) and mean pharmaceutical costs (p = 0.067), between the two groups. According to the bootstrapping procedure, the differences were located within the 95% confidence intervals, for mean total costs [-5826.61, -418.01], mean medication costs is [-93.23, -.57], personnel costs [-4236.76, -282.83] and infrastructure/hotel costs is [-1655.85, -16.0]. According to the paired-sample t-test (e.g. mean total costs, mean medical costs, mean personal costs and mean infrastructure costs), the variable group was significant.

**Fig 2:**
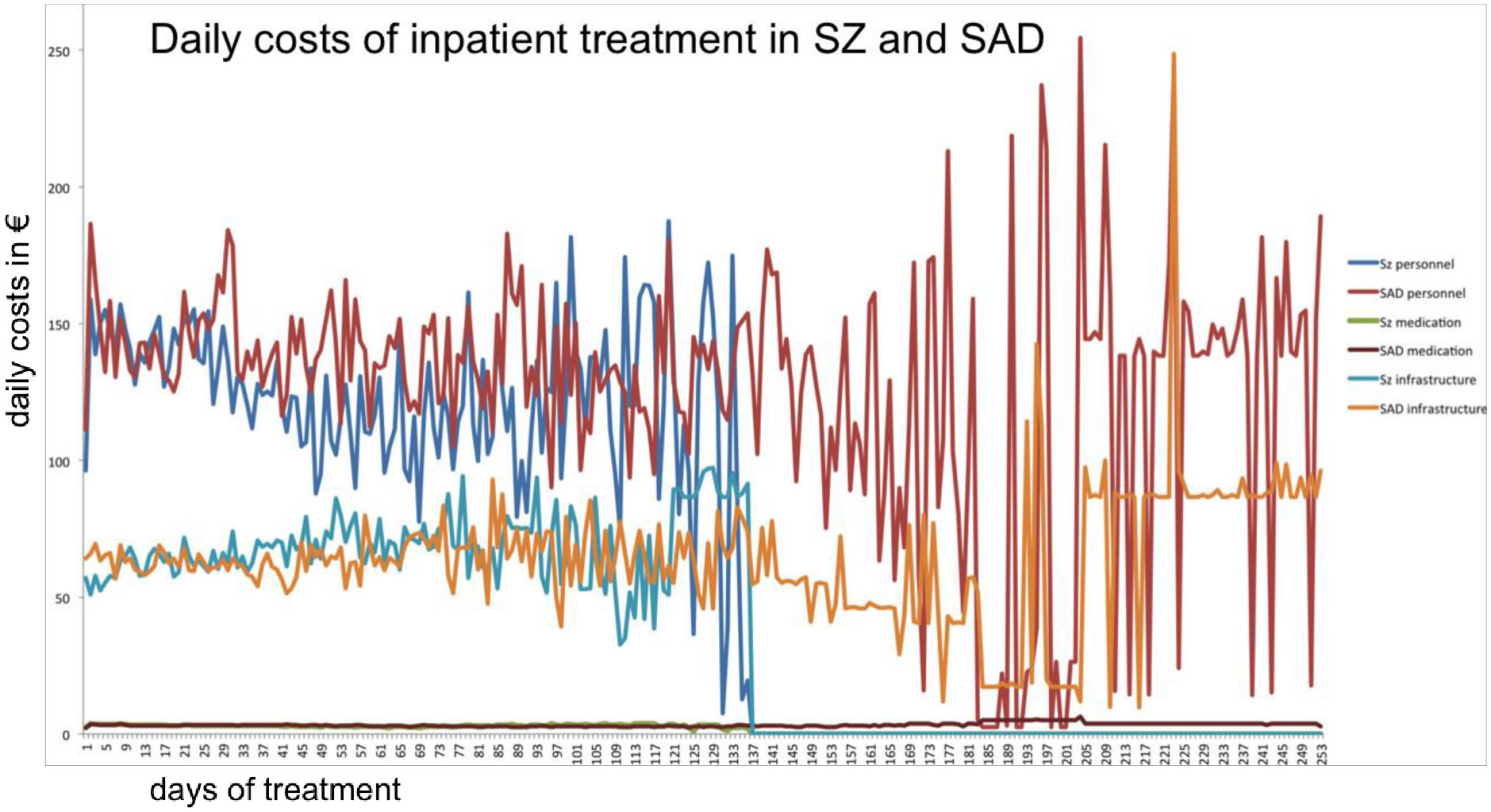
Average daily costs in Euro of inpatient treatment of patients with paranoid SZ (blue-green graphs) and SAD (red-yellow). The timeline does not represent a calendar period of one year but a transformation where all individual treatment courses start at day one of the presented timeline. Note that the number of averaged cases decreases over time. with less than 5 patients received longer treatments than 154 days (i.e. for 167, 180, 183 and 253 days respectively). This results in higher fluctuations of daily means.

In the analysis of group x time effects on costs we found no significant effects within the interval of 8 weeks (Fig 2). In Addition we found no main effect of time in this analysis indicating that there are no significant increments or decrements in the mean total costs, mean costs for medication products, personnel and infrastructure costs as proposed in the national cost accounting system.

## Discussion

To our knowledge this is the first study to compare inpatient health care costs between paranoid SZ and SAD patients in inpatient treatment. The three main findings challenge the current aggregation of both disorders in a single category of resource allocation algorithms: First, SAD patients had a longer duration of stay than patients with SZ. Second, mean total costs were significantly higher for individuals suffering from SAD than paranoid SZ patients. Third, we found we found statistically significant difference in mean personnel costs and a marginal difference in infrastructure costs. In addition there was no evidence for a general or group specific decrement of costs over time.

The extended inpatient period in the SAD group is not surprising for two reasons: First, there is a profound empirical evidence for a lower diagnostic stability of SAD when compared to SZ [20–23]. According to Chen and colleagues [20], the stability rate of SAD diagnosis was 18.6% seven years after the first admission. In two recent longitudinal studies, the stability rate of SAD diagnosis varied between 36% [20] and 73.1% [23]. According to Brenner and colleagues, the main reason for a diagnostic shift are the poor validity and reliability of the SAD diagnosis and the longitudinal course of illness over the years [23]. Along these lines, reaching a reliable diagnosis of SAD appears to be a complex endeavor, that demands more time than diagnosing SZ. A further issue that might extend the duration of stay in the SAD is the severity and the demand to establish a combined and guideline-based treatment of affective and negative symptoms. This is in line with previous studies that showed that SAD patients present with a higher illness severity, mainly due to episodic illness course (episodes including depression, hypomania, and mania) [16,24,25]. In a case of affective symptom fluctuation, a fine-grained adjustment of antipsychotic and mood stabilizing medication might extend the duration of stay in SAD patients.

Contradictory to our assumption and the idea of greater symptom severity in SAD, we found no statistically significant difference in pharmaceutical costs between patients with SZ and SAD. But, we found a statistically significant difference in mean personnel costs between both diagnostic groups. On the one hand, we had still expected a difference in allocated pharmaceutical treatment resources given as SAD patients might show more severe affective and negative symptoms. However, SAD patients also have higher levels of cognitive and social functioning when compared to SZ [26]. These patients’ characteristics in the SAD could account for better integration in a social community and a higher level of social interaction possibly balancing the pharmaceutical treatment costs. On the other hand, psychomotor agitation, aggression and disorganized behavior at the admission might require additional personal involvement and higher costs for combined inpatient treatment needed for SAD patients [27]. These symptoms assumedly cause a higher rate of intensive treatment in acute care wards. This difference in treatment context could also account for differences in infrastructure costs in the treatment of SAD patients.

Another notable issue is the result of repeated measures ANOVA in both patient groups. We found no main effect of time in this analysis indicating that there are no significant increments or decrements in the mean total costs, mean costs for medication products, personnel and infrastructure costs. Based upon these results, we disagree with a previously published recommendation of the “InEK” in Germany to reimburse the hospital costs daily with step-wise decreasing remuneration [28]. This model has been criticized in previous publications [29–31]. Since our data suggest that there is a high risk of inadequate remuneration of SAD patients with longer duration of stay, we object the implementation the cost accounting system in the daily clinical routine. However, there is still a paucity of evidence regarding the exact factors influencing daily treatment resources needed in SAD and paranoid SZ patients.

Finally, we examined the mean total costs between SAD and paranoid SZ. Our results are in line with previous cost-of-illness studies for SZ in Germany [32]. However, to the best of our knowledge, mean total costs for SAD have not yet been investigated by healthcare studies. The higher mean total costs in the SAD group when compared to paranoid SZ group could be a direct consequence of the longer duration of inpatient treatment. While we found no significant difference in the daily pharmaceutical treatment costs between the two diagnostic groups, we suggest that the greatest impact on the mean total costs is generated by the personnel costs required within the inpatient setting. As noted in the introduction, the analysis of the mean total costs might provide complementary information regarding the exact nature of economic impact of SZ spectrum subtypes and improve the distribution of available treatment resources. With caution for the limitations of our study, i.e. small sample size without age-gender-education matching, cross-sectional design, missing information on decreased or increased substance use during treatment, high variance of mean total costs in both patients groups, and a differential demand of resources by paranoid SZ and SAD-specific treatment costs, further studies especially longitudinal assessments of treatment courses and history of diagnoses are needed to substantiate our findings and provide relevant information for healthcare professionals.

## Conclusion

This is the first statistically significant evidence for distinguishable costs among paranoid SZ and SAD patients. The findings of this study provide insights into the economic impact of different nosological entities. Our results may provide important clues for further development of cost accounting systems for reimbursement in Germany, which has subsumed both paranoid SZ and SAD within a single diagnoses related category. In the future, we strongly advocate retrospective audit of data from larger patient samples [31]). Future studies should also compare data of SZ spectrum patients and patients with bipolar disorders.
